# Trans-intervertebral space osteotomy for post-traumatic thoracolumbar kyphosis: a minimum 2-year follow-up study

**DOI:** 10.3389/fneur.2026.1810999

**Published:** 2026-07-03

**Authors:** Yu-liang Sun, Tao Gu, Xuan-Geng Deng, Hua-Gang Shi, Wei Hou

**Affiliations:** Sichuan Provincial Orthopedics Hospital, Chengdu, China

**Keywords:** neurological damages, older patients, spinal deformities, thoracolumbar kyphosis, trans-intervertebral space osteotomy

## Abstract

**Background:**

This study aims to evaluate the radiographic results and clinical efficacy of trans-intervertebral space osteotomy (TIO) in the treatment of post-traumatic thoracolumbar kyphosis (PTK).

**Study design/setting:**

This is a single-center retrospective cohort study.

**Methods:**

A retrospective analysis was conducted on clinical data from PTK patients who underwent TIO between January 2018 and December 2022. A total of 38 patients (18 males and 20 females) with an average age of 52.1 ± 9.2 years were included. According to the Frankel classification, 8 cases were grade D and 30 cases were grade E. Spinal-pelvic sagittal parameters (thoracic kyphosis [TK], thoracolumbar kyphosis [TLK], lumbar lordosis [LL], focal kyphosis [FK], pelvic incidence [PI], pelvic tilt [PT], sacral slope [SS], sagittal vertical axis [SVA]) were measured on lateral spinal X-rays preoperatively, postoperatively, and at the last follow-up. The Visual Analog Score (VAS) and Oswestry Disability Index (ODI) were recorded postoperatively and at the last follow-up, and perioperative/follow-up complications were documented.

**Results:**

The average operation time was 277.4 ± 33.9 min, with an average intraoperative blood loss of 704.5 ± 340.5 mL. All incisions achieved primary healing. The mean follow-up period was 35.1 ± 9.4 months (range: 24–56 months). Postoperatively, TK, TLK, LL, FK, and SVA were significantly improved (*p* < 0.05), while PT, PI, and SS showed no significant changes (*p* > 0.05). These radiographic outcomes remained stable at the last follow-up (*p* > 0.05). The preoperative VAS score (6.0 ± 1.1) decreased significantly to 1.2 ± 0.8 at the last follow-up (*p* < 0.05), and the preoperative ODI (56.9 ± 7.1%) was reduced to 15.7 ± 3.9% (*p* < 0.05). At the final follow-up, the Frankel classification showed 3 cases of grade D and 35 cases of grade E. All fixed segments achieved fusion, with no internal fixation loosening or adjacent vertebral fractures.

**Conclusion:**

TIO for PTK patients achieves satisfactory correction of spinal deformity and favorable clinical outcomes, with successful fusion and few complications.

## Introduction

Post-traumatic thoracolumbar kyphosis (PTK) often develops due to conservative treatment or inappropriate timing/methods of surgical intervention for thoracolumbar fractures, leading to delayed severe kyphosis (1, 2). Thoracolumbar fractures are the most common complications of spinal injuries, often resulting in varying degrees of spinal instability and pseudarthrosis formation (3). Patients with kyphosis have their PTK center of gravity moving forward, causing excessive compressive stress on the anterior column and excessive tensile stress on the posterior column. Prolonged centrifugal loading on the spine leads to wedge-shaped deformities, progressive kyphosis, chronic low back pain, and associated neurological dysfunction (4, 5).

Recently, Wang et al. proposed a classification system for trans-intervertebral space osteotomy (TIO), including three main types and two subtypes (6). Among them, type II TIO is similar to SRS-Schwab grade 4 osteotomy (7), but differs in that it removes adjacent endplates and wedge-shaped bone through the intervertebral space without involving the pedicles. This approach provides a safer osteotomy field with a smaller surgical range, reducing surgical trauma and shortening operation time while achieving effective decompression and correction, demonstrating high safety and efficacy (8, 9). Type II TIO can achieve 20°-25° of deformity correction (10) and is considered an effective surgical option for moderate rounded spinal kyphosis. However, there are limited literature reports on the application of TIO in PTK patients. Therefore, this study retrospectively analyzed the clinical data of PTK patients who underwent TIO combined with internal fixation, aiming to evaluate the radiographic and clinical outcomes of this approach.

## Methods

### Inclusion and exclusion criteria

#### Inclusion criteria

Patients aged 27–70 years; single-segment fractures in the T11–L2 region; disease duration >3 months; spinal kyphosis >30°; refractory lumbosacral pain or progressive neurological damage; follow-up period >2 years with complete clinical and imaging data.

#### Exclusion criteria

Severe underlying medical conditions; pathological thoracolumbar fractures; spinal infections; history of spinal surgery.

### Patients

A retrospective analysis was performed on PTK patients treated at our hospital between January 2018 and December 2022. Based on the above criteria, 38 patients (18 males and 20 females) who underwent trans-intervertebral space osteotomy and internal fixation were included. The age range was 27–70 years, with an average age of 52.1 ± 9.2 years at the time of surgery. Fracture segments were distributed as follows: T11 (5 cases), T12 (15 cases), L1 (12 cases), and L2 (6 cases). The average disease duration was 47.3 ± 40.9 months (range: 6–176 months). All patients presented with preoperative thoracolumbar kyphosis and varying degrees of low back pain, and 8 patients had neurological impairment (8 cases of Frankel grade D and 30 cases of grade E). Preoperative sagittal imbalance was present in 14 patients (sagittal imbalance standard: C7-SVA greater than ±4 cm). The participants signed a written informed consent form before surgery.

### Surgical strategy

Under general anesthesia, patients were placed in the prone position. A posterior midline longitudinal incision was made to fully expose the posterior spinal structures. Polyaxial pedicle screws were inserted at the predetermined fixation segments. An ultrasonic osteotome was used to resect the spinous process and bilateral laminae of the injured vertebra, exposing the dura mater and bilateral pedicles. The lower half of the lamina and the inferior articular process of the proximal vertebra were then removed. On one side, the wedge-shaped upper portion of the injured vertebra and the attached intervertebral disc were resected through the intervertebral space using an ultrasonic osteotome.

After unilateral osteotomy, a temporary rod was used to fix the osteotomy site, followed by contralateral osteotomy using the same technique to resect the corresponding wedge-shaped bone and intervertebral disc. Subsequently, the posterior wall of the vertebral body anterior to the dural sac was removed. Before closing the osteotomy surface, the margins of the upper and lower laminae were slightly trimmed to avoid mechanical compression of the dura mater and nerve roots during correction. Osteotomy fragments from the spinous process and laminae were implanted into the intervertebral space. If the osteotomy gap was too large during closure, an intervertebral fusion cage was placed to avoid spinal cord shortening exceeding 2 cm (30 patients in this group received cage implantation). Finally, bilateral connecting rods were inserted and longitudinally compressed to secure the pedicle screws and correct the kyphotic deformity. If the patient has osteoporosis or more than 5 fused fixed segments, cross-connectors should be used.

After closing the osteotomy surface, the dural sac was carefully inspected for tension, and additional trimming of the upper and lower lamina edges was performed if necessary. The incision was thoroughly irrigated with a large volume of normal saline, followed by posterolateral bone grafting using intraoperative osteotomy cancellous bone and autologous iliac bone. A drainage tube was placed, and the incision was sutured layer by layer. All surgeries were performed by the same group of doctors.

All procedures were monitored by somatosensory-evoked potentials (SEP) and motor evoked potentials (MEP).

### Postoperative treatment

Antibiotics should be used routinely for 48 h following surgery. If the patient continues to have a fever or exhibits high infection indicators, antibiotic use will be extended. The drainage tube was removed when the daily incision drainage volume was <50 mL. Patients were allowed to ambulate with a thoracolumbar brace 3 days after surgery, and brace wear was continued for 3 months.

### Radiographic measurements

Coronal and lateral standard X-rays of the entire spine were obtained preoperatively, postoperatively, and at each follow-up. The measured parameters included (11): (1) thoracic kyphosis (TK); (2) thoracolumbar kyphosis (TLK); (3) lumbar lordosis (LL); (4) focal kyphosis (FK, the angle between the upper lamina of the injured vertebra and the lower lamina of the adjacent upper vertebra); (5) pelvic incidence (PI); (6) pelvic tilt (PT); (7) sacral slope (SS); and (8) sagittal vertical axis (SVA). All radiographic parameters were independently measured by two spine surgeons, and the mean was calculated for analysis.

### Clinical assessment

Operation time, intraoperative blood loss, and surgical complications were recorded. The VAS was used to assess pain intensity, and the ODI was used to evaluate quality of life preoperatively and at the last follow-up. Neurological recovery was assessed using the Frankel classification at the last follow-up. Complications such as internal fixation loosening or breakage were closely monitored, and bone healing and internal fixation-related complications were evaluated by X-rays during follow-up.

### Statistical analysis

SPSS software version 26 (SPSS Inc., Chicago, IL, USA) was used for statistical analysis. All data follow a normal distribution and are presented as mean ± standard deviation (SD). Repeated measures analysis of variance was used to compare radiographic outcomes preoperatively, postoperatively, and at the last follow-up. Paired sample t-tests were used to compare VAS and ODI scores preoperatively and at the last follow-up. Statistical significance was set at *p* < 0.05.

## Results

### General outcomes

The average operation time was 277.4 ± 33.9 min (range: 235–358 min), and the average intraoperative blood loss was 704.5 ± 340.5 mL (range: 300–1,600 mL). The average fusion span was 6.3 ± 1.3 segments (range: 4–8 segments), including 4 cases with 4 segments, 6 cases with 5 segments, 9 cases with 6 segments, 12 cases with 7 segments, and 7 cases with 8 segments. No infection or nerve injury complications occurred. Intraoperative dural tear was noted in 4 patients, and 2 cases of cerebrospinal fluid leakage occurred after repair; however, all incisions achieved primary healing (Table 1). The perioperative complications rate was 10.5%.

**Table 1:** Clinical data of the cohort (*n* = 38).

Parameters	Mean ± SD (range)
Age (yrs.)	52.1 ± 9.2 (27–70)
Disease duration (month)	47.3 ± 40.9 (6–176)
Operating time (min)	277.4 ± 33.9 (235–358)
Blood loss (ml)	704.5 ± 340.5 (300–1,600)
Fusion span (No.)	6.3 ± 1.3 (4–8)
Follow-up (month)	35.1 ± 9.4 (24–56)

### Radiographic outcomes

Preoperative TK was 18.3 ± 4.5°, which increased to 28.2 ± 4.8° postoperatively and remained stable at 28.0 ± 4.6° at the last follow-up. Preoperative TLK was 35.5 ± 5.7°, which decreased significantly to 7.7 ± 2.5° postoperatively and 7.8 ± 2.4° at the last follow-up. Preoperative LL was 60.4 ± 9.9°, which decreased to 44.4 ± 7.8° postoperatively and 44.6 ± 7.8° at the last follow-up. Preoperative FK was 38.9 ± 4.8°, which was corrected to 5.7 ± 2.1° postoperatively and 5.8 ± 2.1° at the last follow-up. PI, PT, and SS showed no significant changes preoperatively, postoperatively, or at the last follow-up (PI: 47.4 ± 7.2° vs. 47.3 ± 7.3° vs. 47.6 ± 7.2°; PT: 18.0 ± 4.2° vs. 18.1 ± 4.3° vs. 18.3 ± 4.6°; SS: 29.4 ± 3.7° vs. 29.2 ± 3.6° vs. 29.3 ± 3.4°). Preoperative SVA was 45.7 ± 11.1 mm, which improved to 16.4 ± 7.1 mm postoperatively and 16.8 ± 6.1 mm at the last follow-up.

**Figure 1:**
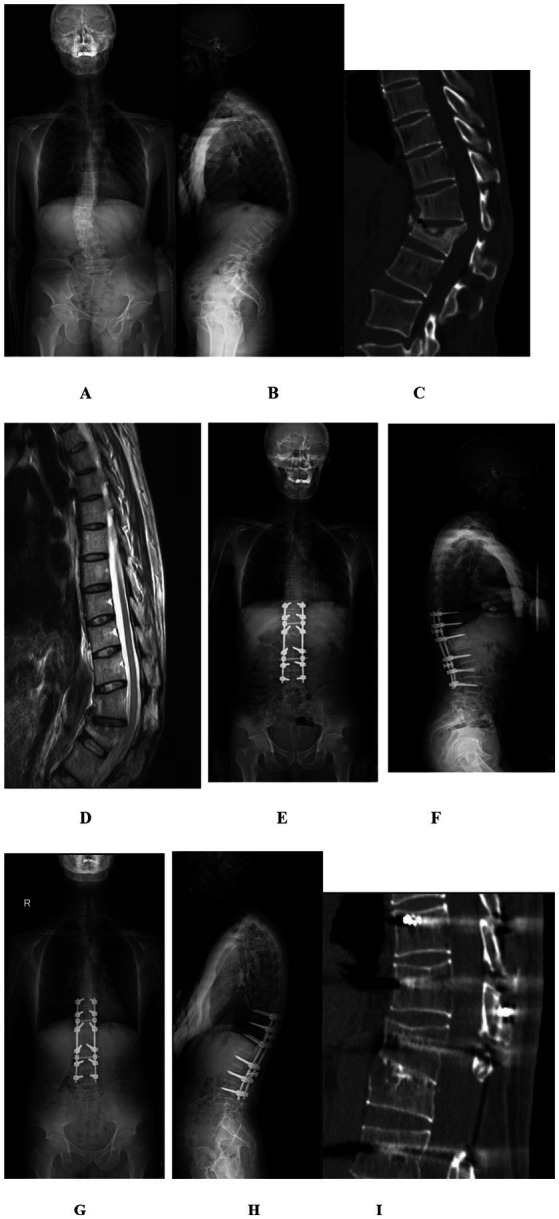
A 51-year-old male with L1 PTK, Frankel grade: grade D **(A–D)**. The patient underwent trans-intervertebral space osteotomy at L1 PTK and fusion from T10 to L4 **(E,F)**. At 26 months follow-up, the patient achieved a solid bone fusion, and the sagittal balance was well maintained, Frankel grade: grade E **(G–I)**.

**Figure 2:**
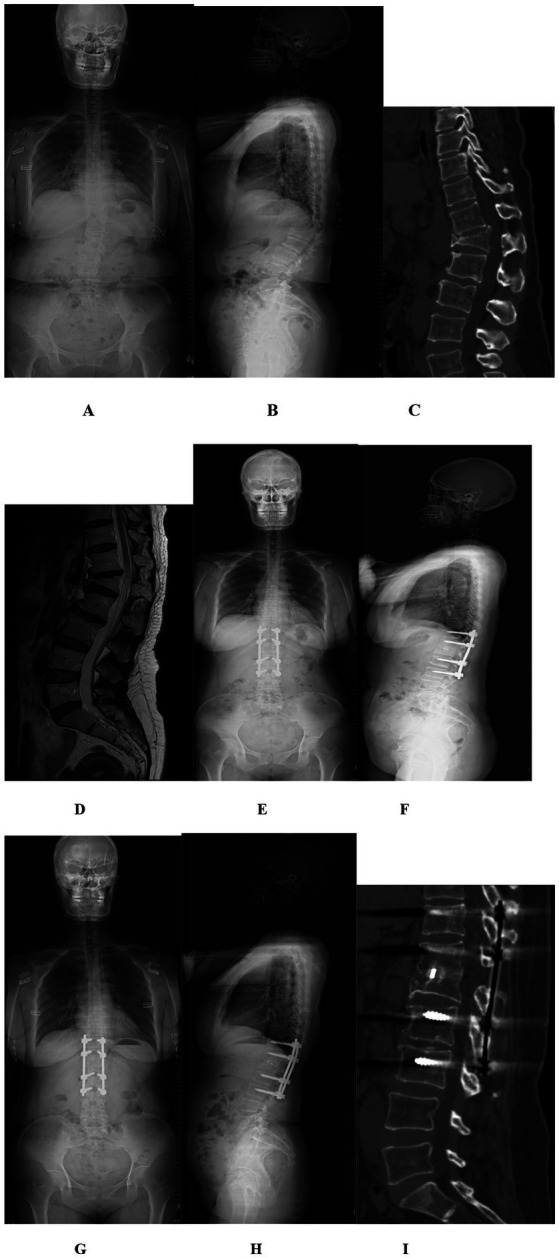
A 55-year-old female with L1 PTK, Frankel grade: grade E **(A–D)**. The patient underwent trans-intervertebral space osteotomy at L1 PTK and fusion from T11 to L3 **(E,F)**. At 36 months follow-up, the patient achieved a solid bone fusion, and the sagittal balance was well maintained, Frankel grade: grade E **(G–I)**.

Compared with preoperative values, TK, TLK, LL, FK, and SVA were significantly improved postoperatively (*p* < 0.05), while PT, PI, and SS showed no significant changes (*p* > 0.05). No significant loss of correction was observed at the last follow-up compared with postoperative results (*p* > 0.05). All patients restored sagittal balance after surgery (Table 2). Representative cases with imaging findings are shown in Figures 1, 2.

**Table 2:** Comparison of radiographic outcomes between pre-operation, post-operation and last follow-up (*n* = 38).

Parameters	Pre-op	Post-op	Last follow-up	*F* value	*p* value	Pre-op vs. Post-op	Pre-op vs. last follow-up	Post-op vs. last follow-up
						*P* value	*P* value	*P* value
TK (°)	18.3 ± 4.5	28.2 ± 4.8	28.0 ± 4.6	312.552	<0.001*	<0.001*	<0.001*	0.198
TLK (°)	35.5 ± 5.7	7.7 ± 2.5	7.8 ± 2.4	768.591	<0.001*	<0.001*	<0.001*	0.378
LL (°)	60.4 ± 9.9	44.4 ± 7.8	44.6 ± 7.8	114.774	<0.001*	<0.001*	<0.001*	0.254
FK (°)	38.9 ± 4.8	5.7 ± 2.1	5.8 ± 2.1	1595.397	<0.001*	<0.001*	<0.001*	0.205
PI (°)	47.4 ± 7.2	47.3 ± 7.3	47.6 ± 7.2	1.262	0.289	0.772	0.103	0.216
PT (°)	18.0 ± 4.2	18.1 ± 4.3	18.3 ± 4.6	0.804	0.452	0.697	0.238	0.361
SS (°)	29.4 ± 3.7	29.2 ± 3.6	29.3 ± 3.4	0.189	0.828	0.634	1.000	0.624
SVA (mm)	45.7 ± 11.1	16.4 ± 7.1	16.8 ± 6.1	237.635	<0.001*	<0.001*	<0.001*	0.537

TK, thoracic kyphosis; TLK, thoracolumbar kyphosis; LL, lumbar lordosis; FK, focal kyphosis; PI, pelvic incidence; PT, pelvic tilt; SS, sacral slope; SVA, sagittal vertical axis. *p < 0.05.

### Clinical outcomes

The preoperative VAS score was 6.0 ± 1.1, which decreased significantly to 1.2 ± 0.8 at the last follow-up (*p* < 0.001). The preoperative ODI was 56.9 ± 7.1%, which was reduced to 15.7 ± 3.9% at the last follow-up (*p* < 0.001). The mean follow-up period was 35.1 ± 9.4 months (range: 24–56 months) (Table 3).

**Table 3:** Comparison of VAS and ODI between pre-operation and post-operation (*n* = 38).

Parameters	Pre-op	Last follow-ups	*t* value	*P* value
VAS (points)	6.0 ± 1.1	1.2 ± 0.8	29.514	<0.001*
ODI (%)	56.9 ± 7.1	15.7 ± 3.9	37.551	<0.001*

VAS, Visual Analog Score; ODI, Oswestry Disability Index. *p < 0.05.

All patients have completed follow-up without loss to follow-up. At the last follow-up, all fixed segments achieved fusion, with no pseudarthrosis or internal fixation-related complications. Neurological function assessed by Frankel classification showed 3 cases of grade D and 35 cases of grade E, with 5 patients with preoperative neurological impairment showing improvement from grade D to E.

## Discussion

According to the AO classification, type B2 fractures are prone to secondary delayed kyphosis due to incomplete rupture of the posterior longitudinal ligament complex (PLC) accompanied by avulsion of the posterior vertebral wall bone. In this study, this subtype accounted for 63.2% (24/38) of surgical cases. The primary goals of surgical treatment for PTK include restoring spinal alignment, correcting kyphosis, recovering spinal cord and nerve function, relieving pain, and preventing deformity progression (12). Several osteotomy techniques are available for kyphosis correction, with most surgeons adopting single-stage posterior osteotomy, such as Smith-Petersen osteotomy (SPO), pedicle subtraction osteotomy (PSO), and vertebral column resection (VCR) (13, 14). However, these techniques have their respective advantages and limitations, and their application in PTK treatment is constrained by various factors (15–18).

SPO is performed by means of V-shaped osteotomy at the thoracolumbar junction along with anterior column distraction, with a relatively constrained corrective angle of only approximately 10°. This surgical procedure is indicated for the correction of kyphotic deformities in which the anterior column retains its load-bearing capacity and the intervertebral spaces preserve a certain range of mobility. Patients with PTK typically present with spinal rigidity; additionally, a subset of these cases may be complicated by spinal stenosis, which subsequently results in nerve root and spinal cord compression. Given that SPO does not confer spinal canal decompression efficacy, this technique often fails to achieve satisfactory corrective outcomes (15). PSO is technically demanding with respect to achieving precise wedge resection at the vertebral body region characterized by a lower anterior margin and a higher posterior margin, while also preserving the superior intervertebral disc. Nevertheless, in the setting of PTK, the superior intervertebral disc is frequently subject to damage or disruption, which hinders the formation of robust bone-to-bone contact between the injured vertebra and adjacent normal vertebral segments, thus entailing a potential risk of long-term fusion failure (16, 17). VCR is indicated for the management of severe kyphotic deformities, yet this approach involves sophisticated surgical maneuvers, substantial surgical trauma, and a high incidence of perioperative complications. Furthermore, the considerable residual space secondary to total vertebral resection necessitates the implantation of titanium mesh or artificial vertebral bodies for spinal reconstruction, which precludes the attainment of “bone-to-bone” contact closure. For these aforementioned reasons, VCR is less commonly utilized in the clinical management of PTK (18).

In 2023, Wang et al. (6) first proposed the concept of TIO, and type II TIO can achieve orthopedic correction of 20 ° ~ 25 °. It achieves bone-to-bone contact on the cutting surface and improves the fusion rate by removing the injured intervertebral disc and the wedge-shaped bone at the posterior edge of the vertebral body. During the operation, the amount of vertebral column osteotomy can be accurately evaluated based on the height difference between the anterior and middle columns, and the bone mass of the injured vertebra can be preserved to the maximum extent during osteotomy correction. Due to the preservation of the injured vertebrae, it can also maximize the protection of blood supply. The pedicle can also serve as a “pivot” for screw rod correction, especially in the operation of correcting kyphosis. By applying downward pressure to the injured vertebra toward the ventral side, the deformity can be corrected to the maximum extent possible. The fixation of the injured vertebra during TIO surgery has achieved a multi-stage anchoring effect, dispersing stress in the injured vertebra area and reducing the probability of loosening and fracture of the internal fixation device after surgery.

In this study, 4 patients experienced intraoperative dural tears, and 2 cases of cerebrospinal fluid leakage occurred after repair; however, all incisions healed successfully. Five patients with preoperative neurological impairment showed improved Frankel grades (from D to E) at the last follow-up. All fixed segments achieved fusion, with no pseudarthrosis or internal fixation-related complications. The VAS score decreased from 6.0 ± 1.1 preoperatively to 1.2 ± 0.8 at the last follow-up, indicating significant relief of lumbosacral pain. The ODI improved from 56.9 ± 7.1% preoperatively to 15.7 ± 3.9% at the last follow-up, reflecting a significant enhancement in quality of life. These results confirm the favorable clinical efficacy of TIO in PTK treatment.

Ma et al. (19) retrospectively analyzed 20 PTK patients who underwent TIO combined with cage implantation, reporting a reduction in Cobb angle from 36.75° preoperatively to 9.30° postoperatively. Liu et al. (20) reported satisfactory outcomes in 2 cases of PTK treated with SRS-Schwab grade 4 osteotomy. Another study by Liu et al. (21) on 42 PTK patients treated with modified SRS-Schwab grade 4 osteotomy showed a decrease in Cobb angle from 38.5° preoperatively to 4.2° 2 weeks postoperatively. Guo et al. (22) used modified bone-disc-bone osteotomy to treat 20 cases of spinal kyphosis, achieving a correction of Cobb angle from 40.2° immediately postoperatively to 9.8° at 24 months. In the present study, FK was corrected from an average of 38.9° preoperatively to 5.7° postoperatively and remained stable at 5.8° at the last follow-up, consistent with the aforementioned studies.

When thoracolumbar fractures result in kyphotic deformity, the trunk center of gravity above the deformity shifts forward. To maintain lower trunk stability, the body compensates through cervical, thoracic, and lumbar curvature adjustments, pelvic rotation, and even knee flexion to shift the center of gravity backward and restore sagittal balance. In this study, TK, TLK, LL, FK, and SVA were significantly improved postoperatively (*p* < 0.05), while PI, PT, and SS showed no significant changes. No significant loss of correction was observed at the last follow-up, indicating that TIO improves thoracic kyphosis and lumbar lordosis, reduces pelvic rotation, and restores sagittal balance by correcting local kyphosis. Preoperative SVA was 45.7 ± 11.1 mm, which improved to 16.4 ± 7.1 mm postoperatively, and all 14 patients with preoperative sagittal imbalance regained balance after surgery.

TIO has obvious advantages in the treatment of PTK, but its surgical indications still need to be controlled. First, intervertebral osteotomy is mainly aimed at the mid posterior column of the spine, and bone grafting through the intervertebral space plays a role in “peak shaving and valley filling.” Therefore, this procedure is mainly suitable for patients with PTK with mild to moderate kyphosis mainly caused by compression of the anterior column. Second, the progressive spinal kyphosis and cobb angle> 30°. Ataka et al. (23) suggest that patients with thoracolumbar kyphosis and a Cobb angle greater than 30 degrees, who experience persistent pain or progressive nerve damage, should consider posterior corrective surgery. Third, patient’s refractory pain the lower back is not effective after conservative treatment, or patient has symptoms of spinal nerve damage. Fourth, pseudoarthrosis formation at the fracture site.

For patients with kyphosis, the selection of fixed segments is particularly important. In this study, a sagittal stabilized vertebra (SSV) was used as the lower fixed vertebra (LIV). An SSV is defined as a vertebra where 50% of its body is located anterior to the posterior sacral vertical line, and it has been proven to effectively reduce distal junctional kyphosis (DJK) (24, 25). For Upper fixed vertebra (UIV) selection, based on the principle of symmetry, the segment at the distal end of the same osteotomy vertebra was fixed.

Intraoperative precautions: (1) The cancellous bone particles implanted in the intervertebral space do not have the ability to support, so the degree of compression should be controlled during the nail rod compression correction operation to prevent excessive compression of the intervertebral space and reduce the risk of neurological complications such as spinal cord shortening and folding. (2) If the osteotomy space is too large when closed, an intervertebral fusion cage was placed to avoid spinal cord shortening exceeding 2 cm. In this patient group, 30 cases involved inserting a cage into the osteotomy site. (3) For patients with osteoporosis, it is crucial to achieve a successful initial attempt during nail implantation to prevent repeated drilling, which can lead to loosening of the nail path. Alternatively, using relatively thick pedicle screws can enhance screw grip, and extend the fixation segment if needed.

This study has certain limitations: it is a single-center retrospective study with a relatively small sample size and insufficient follow-up duration. Long-term outcomes such as correction loss and internal fixation failure require further observation. Future studies with larger sample sizes and longer follow-up periods are needed to validate the findings.

## Conclusion

Trans-intervertebral space osteotomy (TIO) achieves satisfactory correction of spinal deformity and favorable clinical outcomes in PTK patients, with successful fusion and few complications.

## Data Availability

The raw data supporting the conclusions of this article will be made available by the authors, without undue reservation.
